# Low-Carbohydrate, High-Protein, and Gluten-Free Bread Supplemented with Poppy Seed Flour: Physicochemical, Sensory, and Spectroscopic Properties

**DOI:** 10.3390/molecules27051574

**Published:** 2022-02-27

**Authors:** Monika Wójcik, Renata Różyło, Regine Schönlechner, Arkadiusz Matwijczuk, Dariusz Dziki

**Affiliations:** 1 Department of Food Engineering and Machines, University of Life Sciences in Lublin, 28 Głęboka St., 20-612 Lublin, Poland; monika.wojcik@up.lublin.pl; 2 Department of Food Science and Technology, Institute of Food Technology, BOKU—University of Natural Resources and Life Sciences, Muthgasse 18, 1190 Vienna, Austria; regine.schoenlechner@boku.ac.at; 3 Department of Biophysics, Institute of Molecular Biophysics, Faculty of Environmental Biology, University of Life Sciences in Lublin, Akademicka 13, 20-950 Lublin, Poland; arkadiusz.matwijczuk@up.lublin.pl; 4 Department of Thermal Technology and Food Process Engineering, University of Life Sciences in Lublin, 31 Głęboka St., 20-612 Lublin, Poland; dariusz.dziki@up.lublin.pl

**Keywords:** low-carbohydrate bread, poppy seed flour, high-protein bread, ATR-FTIR spectroscopy, crumb texture

## Abstract

Background: This study aimed to determine the effect of poppy seed flour (PF) on the physicochemical and spectroscopic properties of low-carbohydrate, high-protein, and gluten-free bread. Methods: The changes at the molecular level were assessed in bread using attenuated total reflectance Fourier transform infrared spectroscopy (ATR-FTIR). Bread prepared with buckwheat, flaxseed, and pea protein was enriched with PF at a concentration of 5–15%. Results: The results showed that the pasting parameters of dough supplemented with PF were significantly decreased compared to the control sample. The obtained bread samples were characterized by good quality and had 14.6% of carbohydrate, 16.3% of protein, 10.2% of fiber, and 4.0% of fat, with a caloric value of 177 kcal/100 g. The addition of PF had little influence on crumb mechanical properties. The ATR-FTIR analyses revealed spectral changes in the region related to protein and carbohydrate structures, as well as changes in band intensity characteristic of α-1,4-glycoside and α-1,6-glycoside bonds. The analyses showed that the main starch skeleton remained clearly visible. Conclusions: PF up to 10% can be potentially applied as a functional ingredient in the production of bread based on buckwheat and linseed flour. Such low-carbohydrate bread can be particularly useful to diabetics.

## 1. Introduction

The rise of several civilization diseases necessitates the development of novel functional bread. In recent years, there has been a lot of focus on developing gluten-free bread recipes; researchers are attempting to produce high-protein bread [1,2], with low-carbohydrate bread receiving less attention [3,4]. 

Poppy seeds are one of the world’s most important and oldest oil crops. They contain a high amount of K, Ca, and P (V) ions. This distinguishes poppy seeds as a valuable dietary supplement with potential for use in the production of novel food products. Moreover, research has shown that the content of Cu (II), Zn (II), Pb (II), and Hg (II) ions in the poppy seeds does not exceed the maximum allowable concentration. This signifies that poppy seeds are safe for consumption [5].

Although poppy seed is rich in nutrition, studies on it as a food additive are scarce. Only some researchers have attempted using poppy seeds to produce biscuits [6] or burgers [7]. In biscuit production, the recipe was supplemented with defatted poppy seeds at an amount of 5%, which resulted in an increase in phenolic content in the final product. The seeds were also found to be rich in tocopherols, which exhibit strong antioxidant properties [6]. 

Poppy seeds are also used as a topping for bread [8]. In some studies, either whole poppy seeds or poppy seeds mixed with other seeds were added to buckwheat bread [9] or gluten-free bread [8,10]. The authors of the studies reported that buckwheat bread enriched with poppy seeds or with other seeds and nuts is an excellent source of selected macro- and microelements and contains bioactive substances, such as phenols, essential oils, unsaturated fatty acids, fiber, and vitamins. 

Thus far, changes in the molecular structure of bread caused by supplementation with various additives, including poppy seeds, have not been investigated in any study. It has been indicated that addition of different food supplements, on the one hand, caused changes in the chemical, mechanical, sensory, and physical properties of starch and, on the other hand, increased the scope of its functional applications. However, it must be emphasized that such practices as modification and supplementation in food processing techniques can cause disruption or changes in certain molecular structures due to, for example, interactions between particular ingredients. This, in turn, can have undesirable effects on the physicochemical, mechanical, and organoleptic properties of food [11]. Hence, all aspects of modifications should be studied by researchers and producers before their incorporation in the food production process, in order to develop the best and safest possible products. For this reason, studies tend to increasingly apply Fourier transform infrared spectroscopy (FTIR) which is a highly noninvasive, fast, and effective method of spectral analysis [11,12,13]. 

Starch is composed mainly of two types of α-glucans, namely amylopectin and amylose, which are connected by α-1,4-glycoside or/and α-1,6-glycoside bonds. These glucans are characterized by a crystalline/amorphous structure, with the short-range order particularly corresponding to the double- and single-helical amylose or amylopectin, which is located in amorphous or crystalline lamellar regions, as well as amylose–amylopectin helix complexes [11]. This clearly suggests that starch microstructures and conformations undergo modifications during processing or supplementation and/or that the amount of water absorbed by the microstructures has changed, which alters the ratios of crystalline/amorphous regions of starch as well as its susceptibility to enzymatic hydrolysis, texture, and/or sensory or rheological properties.

Literature reports indicate that attenuated total reflectance Fourier transform infrared spectroscopy (ATR-FTIR) has been used to assess the most characteristic infrared bands of starch, and the folding of amylose and amylopectin chains. A study assessed the retrogradation of starch using FTIR and concluded that the spectral region of, for example, 800–1100 cm^−1^ contained sensitive bands related to the conformation of the starch polymer (skeleton related to vibrations in α-1,4-glycoside and α-1,6-glycoside bonds, –CH2 vibrations, etc.) and can, therefore, be taken into account in the investigation of, among others, crystallite fusion and multistage retrogradation processes [11]. Other studies confirmed that the correlation between the absorbance intensity of bands identified at approximately 1047 and 1022 cm^−1^ may be considered in the quantitative determination of the crystallinity index, as those bands may be associated with the respective ordered and amorphous structures within the given food material. 

No study has, so far, analyzed the enrichment of high-protein and low-carbohydrate bread with poppy seed flour (PF). Therefore, the present work analyzed the rheological properties of PF-supplemented dough, as well as the physical and antioxidant properties of high-protein and low-carbohydrate bread enriched with PF. In addition, the effect of supplementation of PF at various amounts on changes in the molecular properties of the obtained product was determined by a spectroscopic analysis of FTIR bands. The findings of this study could contribute to expanding the knowledge on the impact of PF on starch at the molecular level and the resulting conformational changes in the given product.

## 2. Results and Discussion

### 2.1. Pasting Behavior of Bread Mixtures

**Figure 1:**
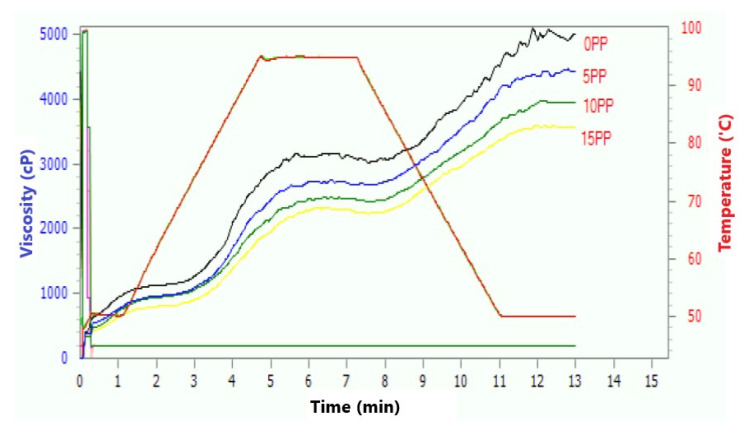
Pasting properties of studied flour blends with different addition of poppy flour: C–control sample, 5PF—blend of flours with 5% poppy flour added, 10PF—blend of flours with 10% poppy flour added, 15PF—blend of flours with 15% poppy flour added.

The typical pasting curves of flour samples supplemented with PF, which were generated based on RVA, are shown in Figure 1 and Table 1. The highest peak viscosity value was observed for the control sample (an aqueous mixture of buckwheat and flaxseed flour with pea protein, psyllium husk, potato fiber, and guar gum). The mixture of buckwheat and linseed flour with pea protein exhibited higher swelling ability, which was probably related to the weaker bonding forces between them.

On the other hand, the high-protein defatted PF displayed lower swelling ability due to the stronger bonding between the ingredients and, thus, exhibited lower peak viscosity [14]. It should also be mentioned that the starch content of the resulting blend was decreased by the addition of PF, which contributed to lower viscosity. The results showed that the peak viscosity decreased with the increase in the amount of PF added (466, 239, and 137 cP for 5%, 10%, and 15% of PF, respectively). Similarly, trough, setback, and final viscosity were found to be the highest for the control sample. This is in line with the results of Alvarez-Jubette et al. [15] who reported that the values of breakdown viscosity were considerably lower for the gluten-free bread prepared using pseudocereal (e.g., amaranth, quinoa, or buckwheat) flours compared to bread prepared with rice flour. Regarding pasting temperature, it was found that the PF as a component of the mixture did not cause any change in this parameter.

### 2.2. Physical Properties of Bread

The moisture level of the obtained breadcrumbs remained the same at about 55% (Table 2). A slight decrease in baking loss from 14.5% (control bread) to about 13.0% was observed with the addition of PF, but this change was not influenced by the amount of PF added. It was also noticed that loaf volume significantly increased with the addition of PF, with the highest volume observed with 10% of this flour (Table 2). Addition of 15% of PF caused a decrease in loaf volume, but the value of loaf volume of bread enriched with 15% PF was statistically significantly higher compared to that of the control bread. This was probably due to the higher protein content of PF. Gluten-free bread usually has a low volume; therefore, use of additives is critical to increase its volume [16,17]. Enrichment with PF also caused a slight but statistically significant decrease in the pH of crumb in low-carbohydrate and high-protein bread; the crumb of bread loaves supplemented with PF had a similar pH at the level of 5.3. 

The crumb of the control bread appeared dark brown in color. This could be due to the darker color of flaxseed flour, which resulted from the presence of a pigment in the outer seed coat (Table 2). The addition of PF did not cause any visual change in the color of the crumb, as was confirmed by the lack of significant differences between the ∆E* values of analyzed breadcrumbs. Only a slight decrease in brightness from 41.7 (control bread) to 39 was observed with the highest proportion of PF. However, no significant differences were noticed in the remaining color parameters, such as a*, C*, and h° values. Regarding the b* value, which indicates blueness/yellowness, a decrease was observed only in relation to the crumb with the highest amount of PF (from 20.1 to 19.2). The darker (browner) color of this type of bread attracted consumers’ attention. Similarly, the addition of ground flax seeds resulted in a favorable darker color in gluten-free bread [17]. In the case of linseed oil, color has been shown to be contributed by carotenoids (i.e., β-carotene) [18]. 

### 2.3. Texture Parameters of Bread

**Figure 2:**
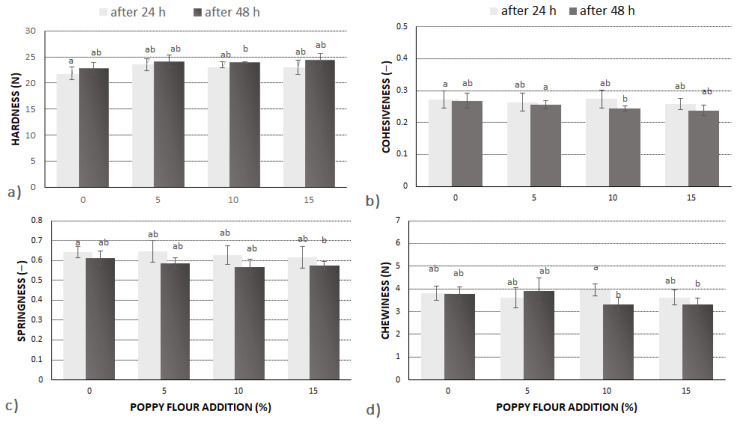
Crumb texture properties of low-carbohydrate and high protein bread supplemented with poppy flour (PF). Values with different letters are significantly (α = 0.05) different. (**a**) Hardness, (**b**) Cohesiveness, (**c**) Springiness, (**d**) Chewiness.

The crumb of the bread enriched with PF was characterized by a higher hardness value, but the increasing share of PF had no significant impact on this parameter (Figure 2). Regarding cohesiveness, addition of PF above 5% resulted in a slight decrease in value. On the other hand, springiness of all tested samples was found to be similar after 24 h of storage. In the case of bread enriched with PF, prolonged storage (48 h after baking) caused a slight decrease in springiness to 0.57. Chewiness of the breadcrumbs supplemented with PF was also found to be similar to the control sample (above 3 N) after 24 h of baking. However, after 48 h, a slight decrease of chewiness (by about 0.4 N) was noted in breadcrumbs with 10% and 15% of PF. Gluten-free bread is usually characterized by high hardness, low elasticity, and low springiness [17]; thus, it is important to ensure that these parameters are not worsened further. A base recipe for gluten-free bread with a low amount of carbohydrates and a high amount of protein was formulated in a previous work [19], which was also accepted by consumers. The results of the present study confirmed that the addition of PF did not cause any adverse textural changes in bread.

### 2.4. Sensory and Calorific Value of Bread

**Figure 3:**
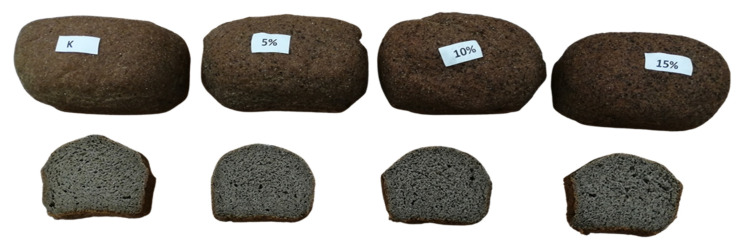
Overall view of low-carbohydrate bread supplemented with poppy flour.

Samples enriched with 5% and 10% of PF were rated the best by evaluators (Table 3). These were given higher scores for features such as taste, smell, and color. On the other hand, the control sample was rated as poor in terms of taste and smell. The dark color of the low-carbohydrate bread crumb was positively perceived by the panelists as opposed to its texture, which was evaluated as the poorest of all the assessed features (Figure 3). Bread containing the highest amount of PF received the lowest scores for quality parameters such as taste, texture, and overall acceptability. It had a slightly bitter aftertaste, and the texture was slightly crumbling. The bitter taste of the bread containing the highest proportion of PF was probably related to the highest amount of oxylipins [20]. Thus far, only two studies have conducted a similar sensory analysis of buckwheat bread prepared with poppy seeds added at a range of 2.2–7.4% [9].

Supplementation with PF changed the chemical composition of low-carbohydrate bread (Table 3). Enrichment of bread with 10% of PF caused a decrease in the content of fiber (by 2%), carbohydrates (by 2.3%), and protein (by <1%) in comparison to the control sample. On the other hand, a higher fat content of 4% was noted (an increase of 0.7%). The obtained low-carbohydrate bread with high protein content was characterized by a lower caloric value of 177 kcal/100 g. Świeca et al. [9] also obtained gluten-free bread enriched with milk and poppy seeds, which had a slightly higher nutritional value (191 kcal) as comparable to the control sample studied herein. 

### 2.5. TPC and AA of Bread

TPC and AA analyses showed that the lowest TPC was found in PF (0.42 ± 0.06 mg GAE/g d.m.) and the highest in the control flour (1.45 ± 0.13 mg GAE/g d.m.). This suggests that addition of PF in the recipe decreased the TPC of bread (Table 4). Ishtiaque et al. [21] studied different oil seeds and found that PF is a relatively poor source of polyphenols. However, significant differences in TPC were observed only between bread containing 15% of PF and samples containing 0% and 5% of PF. AA, which was determined by ABTS and DPPH assays, was also found to be decreased with PF enrichment. The results showed that PF had several-fold lower TPC and AA in comparison to control flour. The control flour used was a mixture of buckwheat flour and flaxseed flour. Both these ingredients are characterized by high antioxidant capacity and are commonly used for food fortification [22]; even after the addition of PF, the overall AA of these ingredients remained high.

### 2.6. Amino Acid and Fatty Acid Composition of Low-Carbohydrate Bread with Optimal PF Supplementation

Enrichment of low-carbohydrate bread with 10% of PF caused an increase in the content of analyzed amino acids including asparagine, serine, glutamic acid, proline, alanine, leucine, and valine (Table 5).

The analysis of fatty acid content showed a significant increase in the amount of total fatty acids, especially palmitic acid and linoleic acid + *trans*-9,12-octadecadienoic acid in bread samples fortified with 10% of PF (Table 6). Previous studies [23] have revealed that linoleic acid was dominant in all the analyzed poppy oils. The other major fatty acids in these oils were palmitic and oleic acids, while stearic acid, alpha-linolenic acid, and palmitoleic acid were found in minor amounts. All these fatty acids are considered valuable due to their ability to confer protection against cardiovascular disease, heart attacks, and many inflammatory diseases.

### 2.7. Characteristics and FTIR Analysis of Changes in the Spectra of the Studied Bread Samples

**Figure 4:**
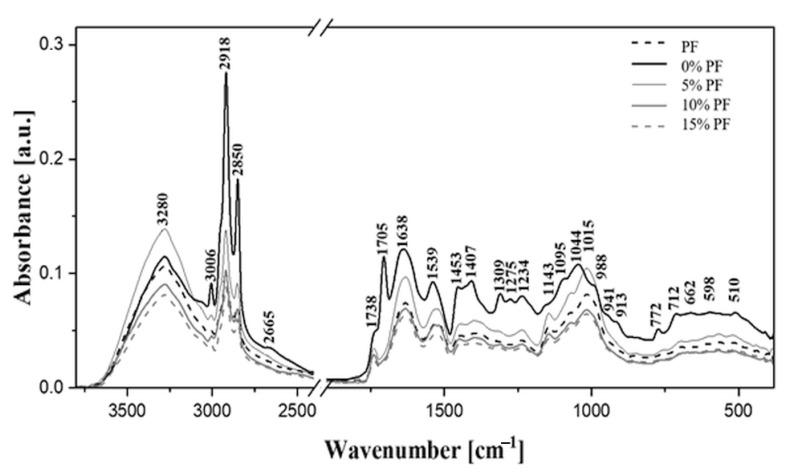
ATR/FTIR spectra of the analyzed samples taken in the spectral range from 450 to 3750 cm^− 1^. Respectively: dashed black line–poppy flour (PF), heavy solid black line–control bread without PF, fine solid black line–5% PF, heavy solid grey line–10% PF, dashed grey line–15% PF. All the spectra were registered at room temperature.

A detailed analysis of the samples of bread containing PF at the molecular level was carried out using ATR-FTIR. For the purpose of discussion and clear presentation of results, all the relevant spectra are shown in Figure 4 and the corresponding types of vibrations are illustrated in Table 7.

Based on a detailed review of literature, including the studies by Al-Mahsaneh et al. [12], Dankar et al. [11], and Sivam et al. [13], it was identified that the first characteristic region of vibrations observed in the spectra (Figure 4), i.e., the range with the maximum at approximately 3280 cm^− 1^, corresponds to the stretching vibrations of –OH groups present in the starch structure. With the addition of increasing amount of PF, a clear decrease in the band’s intensity was noted, which suggests the higher share of vibrations originating from hydrogen bonds with the increased content of PF. Another important area relates to the stretching vibrations of –C–H groups, specifically the –CH2 groups present in the structure of starch and the additives used [11,13]. The maxima of the vibrations were identified at approximately 3006, 2918, and 2850 cm^−1^. It should be noted that, with increasing supplementation of PF, a significant decrease was observed in the intensity of vibrations, which may be associated with the decreasing share of carbohydrates in the resulting product. In turn, deformation vibrations of hydroxylic groups were noted as a band with the maximum at approximately 1638 cm^−1^ (Figure 4) [24]. It seems, however, that the characteristic vibrations of the amide I grouping, which is a key component of the secondary protein structure in the samples, might have made a significantly higher contribution to the intensity of this band [11]. The amide I band in this study corresponded to the stretching vibrations of C=O and C–N groups, as well as the deformation vibrations of the N–H group [11,13]. The characteristic band at approximately 1738 cm^−1^ was associated mainly with the stretching vibrations of the carbonyl group [11,24], with the highest intensity thereof recorded for the flour containing no poppy and lower intensity recorded for the remaining samples. In the case of the sample not containing poppy, this effect was mainly an enhancement of the very intensive band with the maximum at 1705 cm^−1^, which was not found in the spectra obtained for samples containing PF. With the addition of increasing amounts of PF, the intensity of this band rapidly deteriorated. This may be attributed to the formation of hydrogen bonds between the primary units in the structure of the main ingredient, caused by the increasing addition of PF. 

The analysis of further spectral regions, particularly the fingerprint region, also provided a lot of interesting information. Firstly, it must be mentioned that the first characteristic band with the maximum at approximately 1539 cm^−1^ was associated with the vibrations of the amide II grouping [11,13]. In this case, these vibrations refer to the deformation vibrations of N–H, stretching vibrations of C–N, and stretching vibrations of C=O groups. Next, the vibrations with the maxima at 1453 and 1407 cm^−1^ are characteristic of proteins [13,25]. The band with the maximum at approximately 1407 cm^− 1^ can be related to the characteristic deformation vibrations of the –CH2 group and stretching vibrations of the –COO group. In addition, there were other important bands with the maximum at approximately 1234 cm^−1^, which are characteristic of proteins, i.e., vibrations of the amide III grouping, mainly stretching vibrations of C–N, deformation vibrations of N–H, and stretching vibrations of C–C groups. The vibrations with the maxima at approximately 1143, 1095, 1044, and 1015 cm^−1^ are characteristic of, respectively, stretching of C–O, C–C, and C–O–C groups. These correspond to the crystalline and amorphous regions of starch [11,13]. The two first maxima are stretching vibrations that are characteristic primarily of polysaccharide molecules [13]. Addition of PF significantly influenced the maxima locations for the bands in this region. Remarkable changes, especially in the intensity of bands, were also recorded in the spectral range of 945–470 cm^−1^. The bands are mainly associated with the vibrations of the sugar fraction present in the main ingredient in the samples [11,12,13]. The observed changes were significant in almost the entire range, although they mainly concerned the band intensity. The vibrations that are characteristic of the groups in this region correspond mainly to the hydrogen bonds formed between starch units and the α-1,4-glycoside and α-1,6-glycoside bonds between monomers in the structure of the main ingredient [11,13,24]. 

In summary, infrared spectroscopy allows further research in the areas of enrichment of bread with PF additives and, thus, can be particularly useful in evaluating the quality of healthy food. It is important to note the changes in the intensity of bands throughout the spectral region. Furthermore, one can observe clear shifts in the registered spectra in certain spectral ranges, which depend on the amount of additives added, as well as the emergence or disappearance of certain bands after the addition of additives. 

## 3. Materials and Methods

### 3.1. Materials

Buckwheat flour (Helcom, Cracow, Poland), flaxseed flour (BioPlanet, Leszno, Poland), PF (Efavit, Poznań, Poland), and pea protein powder (BioPlanet, Poland) were purchased from health food stores. The composition of buckwheat flour, flaxseed flour, and PF was, respectively, 13.1%, 40.1%, and 36% of protein (ISO 20483:2006) [26]; 3.2%, 8.9%, and 19.1% of fat (ISO 11085:2008) [27]; 63%, 4%, and 19% of carbohydrates (estimated by subtraction); 4%, 33.8%, and 10.5% fiber [28]; and 1.3%, 6.8%, and 6.4% of ash content (ISO 2171:2007) [29]. Additionally, high-protein pea powder (BioPlanet, Poland) having a protein content of 78% was used in the recipes. Psyllium husk (Dimica, Bratislava, Slovakia), potato fiber (Spiegelhauer, Bremen, Germany), guar gum (Nat Vita, Długołęka, Poland), dried yeast (Lesaffre, Maisons-Alfort, France), and Himalayan salt (Intenson, Karczew, Poland) were also added.

### 3.2. Rapid Visco Analyzer (RVA) Measurements

The effects of PF on the pasting properties of bread were investigated using an RVA-4500 system (Perten Instruments, Macquarie Park, Australia), according to the standard method 22–08 (AACCI, 2000), as described by Wójcik et al. [19]. This analysis was carried out in triplicate. 

### 3.3. Breadmaking Procedure

The control bread samples were prepared based on a previous study [19], with the following ingredients: 45 g of buckwheat flour, 45 g of flaxseed flour, 10 g of pea protein powder, 4 g of psyllium husk, 2 g of potato fiber, and 2 g of guar gum. In addition, 1 g of dried yeast, 2 g of Himalayan salt, and 130 mL of tap water were used for making bread. The base recipe of the bread was supplemented with PF in the proportions of 5%, 10%, and 15%, instead of buckwheat and flaxseed flour, respectively. All ingredients were added and mixed using a spiral mixer (CLATRONIC KM 3630, Opole, Germany) for 2 min at 100 rpm and for 4 min at 200 rpm. After mixing, the dough was divided into portions (120 g), gently rounded, placed on loaf tins (95 mm × 60 mm top; 80 mm × 50 mm bottom), and then fermented for 60 min (30 °C and 80% relative humidity). Next, the fermented dough pieces were baked using a laboratory oven (Sadkiewicz Instruments, Bydgoszcz, Poland) for 25 min at 210 °C. Baking was carried out in three replicates. Finally, the bread loaves were cooled to room temperature, packed in polyethylene bags, and stored for 24 h before they were subjected to testing.

### 3.4. Analysis of Physical Properties of Bread

Using the millet seed displacement method, the volume of bread loaves was measured [19], and the obtained results were converted to 100 g of bread. The pH of the bread crumb was determined using a 206-ph2 pH meter (Testo, Pruszków, Poland). Immediately after baking, the bread samples were weighed to calculate the baking loss, which was expressed as a percentage of weight loss derived by subtracting the mass of bread from that of dough and then dividing by the mass of the dough. Bread volume and baking loss measurements were made in triplicate.

The color of bread crumb was determined in CIE L*a*b* system using a CR30-16 colorimeter (Precise Color Reader, 4Wave, Tychy, Poland) by assuming the following parameters: illuminant D65 and 10° standard observer. In the CIE L*a*b* color system, L* indicates lightness/darkness (from 0 to 100), a* denotes the degree of redness (+)/greenness (−), and b* represents the degree of blueness (−)/yellowness (+). Measurements were done in triplicate.

### 3.5. Texture Evaluation of Bread

The texture of bread crumb was assessed using a ZWICK Z020/TN2S testing machine (Zwick Roell Group, Ulm, Germany) after 24 and 48 h of baking. For the purpose of measurement, the bread sample (20 mm × 20 mm) cut from the central part of the slice (14 mm thickness) was double-compressed to 60% of its thickness, at a speed of 20 mm s^−1^. Curves were plotted using the thickness values of bread crumb with testXpert V 7.1 simulation software (Zwick Roell Group, Ulm, Germany) of the testing machine. Based on the curves, parameters such as hardness, cohesiveness, springiness, and chewiness [30] were determined. The measurement of each parameter was made in eight replicates.

### 3.6. Sensory Evaluation and Calorific Value of Bread

The sensory properties of low-carbohydrate bread were evaluated by 56 panelists (21–70 years, 30 females and 26 males). During the evaluation, attributes such as color, taste, odor, texture, and overall acceptability of bread were assessed using a 7-point ranking scale [31]. The points on the ranking scale indicated the following: 7—like very much, 6—like moderately, 5—like slightly, 4—neither like nor dislike, 3—dislike slightly, 2—dislike moderately, and 1—dislike very much.

### 3.7. Amino Acid and Fatty Acid Profile Analysis

The amino acid composition of bread samples was determined by acid protein hydrolysis as described by Davis and Thomas [32]. The content of sulfur amino acids and tryptophan was estimated as proposed by Schramm et al. [33]. The concentrations of other amino acids were analyzed using an AAA 400 acid analyzer (Ingos, Prague, Czech Republic) as described by Ziemichód et al. [34]. Analyses were done in triplicate.

The fatty acid composition of the low-carbohydrate bread was determined based on their corresponding methyl esters, which were prepared according to the ISO 12966-2:2017-05 method [35]. Analysis was performed using a Varian 450-GC gas chromatograph (Chrompack, Middelburg, The Netherlands) equipped with a Select™ Biodiesel capillary column (30 m length × 0.32 mm i.d., 0.25 μm film thickness). Galaxie™ Chromatography Data System software (Varian, Mitchell Drive Walnut Creek, CA, USA) was used to control the chromatograph, as well as collect, integrate, and recalculate the results.

### 3.8. Total Phenolic Content (TPC) and Antioxidant Activity (AA)

For determining the TPC and AA of control flour, PF, and enriched bread samples, their methanolic extracts were prepared. Briefly, 0.5 g of each sample was extracted with 5 mL of methanol using the procedure of Romankiewicz et al. [36]. The extracts were shaken for 30 min and centrifuged (13,000× *g*, 10 min). The supernatants were collected and used for biochemical analyses by following Folin–Ciocalteu’s method [37] with slight modifications. 

The AA of OH extracts was assessed by determining their radical-scavenging activity against stable DPPH and ABTS radicals as described by Brand-Williams et al. [38] and Re et al. [39], respectively. The blank samples used for the analysis contained 50% methanol instead of the extract. The following equation was used for assessment:

Scavenging % = [(AC − AA)/AC)] × 100(1)

where AC is the absorbance of the control and AA is the absorbance of the sample.

The half-maximal inhibitory concentration (EC50) or the concentration at which the tested compound showed 50% of the maximum inhibition was calculated in fitted models, based on dose-dependent mode of action [36].

### 3.9. Infrared Spectra Measurements 

The infrared spectra of selected samples of control bread and PF-enriched bread were obtained using an IRSpirit spectrometer (Shimadzu, Japan). For the purpose of measurements, an ATR attachment, i.e., a ZnSe crystal with adequate geometry (truncated at 45 o), was used to ensure 20-fold internal reflection of the laser beam. For each sample, a total of 24 scans were registered. After measurement, the software automatically averaged the results. Before and after each measurement, the crystal was carefully cleaned using ultraclean solvents (Sigma-Aldrich, Poznań, Poland). Exactly 1 h before and continuously during each measurement, a neutral (N2) atmosphere was maintained inside the chamber. All the spectra were registered at room temperature within the range of 450–3800 cm^−1^ at an excellent resolution of 1 cm^−1^. Measurements were performed at the Laboratory of the Department of Biophysics, University of Life Sciences in Lublin. Before the analysis, each spectrum was processed using Grams AI software (Thermo Galactic Industries, Waltham, MA, USA). 

### 3.10. Statistical Analyses

All statistical analyses were performed using Statistica 12.0 software (StatSoft, Cracow, Poland), and differences were considered significant at a level of α = 0.05. Each parameter was evaluated using a one-way analysis of variance (ANOVA). If ANOVA showed significant differences, the means were compared by Tukey’s range test.

## 4. Conclusions 

The results confirmed that PF can be potentially applied as a functional ingredient in the production of bread based on buckwheat and linseed flour. Such low-carbohydrate bread can be particularly useful to diabetics. However, the concentration of PF added should not exceed 10% because it may cause a bitter aftertaste. The study showed that optimal PF supplementation resulted in breads with good quality parameters. Compared to the control bread, bread enriched with 10% of PF had a higher loaf volume. In addition, PF supplementation did not cause adverse textural changes. Enrichment of bread with PF also increased the content of valuable amino acids including asparagine, serine, glutamic acid, proline, alanine, leucine, and valine as well as the total amount of fatty acids. Moreover, PF-enriched bread had a reduced caloric value.

FTIR analysis revealed that the main starch skeleton in the end product (formed by amylase and/or amylopectin) remained visible. However, the addition of PF clearly affected the positions of certain spectral regions as well as their intensity/spectral shift. The changes observed in protein and carbohydrate content in the end product clearly corroborated with the results reported in other studies. Additives can react strongly with starch at the molecular level, leading to the cleavage of hydrogen bonds and consequently alterations in the conformation of starch. They also infiltrate the molecules of the main starch components and, with increasing concentration, can influence their structures, thereby altering them directly or stimulating them to assume more ordered forms. Changes in starch structure can affect the quality, volume, and texture of bread; however, this warrants more research. In the subsequent papers dedicated to this topic, these problems will be explored in detail by extensive spectral analyses.

## Figures and Tables

**Table 1:** Viscosity values of studied flour blends with different addition of poppy flour.

Sample	Peak Viscosity (cP)	Trough Viscosity (cP)	Breakdown Viscosity (cP)	Final Viscosity (cP)	Setback Viscosity (cP)	Peak Time (min)	Pasting Temperature (°C)
C	3177 ± 53 ^a^	2976 ± 45 ^d^	200 ± 20 ^a^	4813 ± 68 ^a^	1837 ± 44 ^a^	6.10 ± 0.83 ^a^	50 ± 0.05
5PF	2711 ± 62 ^b^	2639 ± 37 ^c^	72 ± 15 ^b^	4361 ± 61 ^b^	1722 ± 46 ^b^	6.56 ± 0.27 ^b^	50 ± 0.10
10PF	2574 ± 63 ^c^	2496 ± 48 ^b^	78 ± 11 ^b^	3989 ± 28 ^c^	1493 ± 40 ^c^	6.22 ± 0.21 ^a^	50 ± 0.11
15PF	2355 ± 45 ^d^	2278 ± 39 ^a^	77 ± 17 ^b^	3607 ± 58 ^d^	1329 ± 32 ^d^	6.62 ± 0.26 ^b^	50 ± 0.12

^a^, ^b^, ^c^, ^d^—Values in the same column with different letters are significantly (α = 0.05) different. C–control sample, 5PF—blend of flours with 5% poppy flour added, 10PF—blend of flours with 10% poppy flour added, 15PF—blend of flours with 15% poppy flour added.

**Table 2:** Physical properties of low-carbohydrate and high protein bread supplemented with poppy flour (PF).

Properties of Bread	Control Bread (0%PF)	Bread with 5% PF	Bread with 10% PF	Bread with 15% PF
Moisture of Bread (%)	55.1 ± 0.22	54.8 ± 0.34	55.1 ± 0.23	55.4 ± 0.33
Baking Loss (%)	14.8 ± 0.45 ^b^	13.1 ± 0.48 ^a^	13.3 ± 0.33 ^a^	13.6 ± 0.49 ^a^
Volume of 100 g of Bread (cm^3^)	145.5 ± 1.82 ^a^	147.6 ± 0.93 ^a^	155.7 ± 0.44 ^c^	153.1 ± 1.67 ^b^
pH (−)	5.4 ± 0.03 ^a^	5.3 ± 0.02 ^b^	5.3 ± 0.02 ^b^	5.3 ± 0.02 ^b^
Color L* Value	41.7 ± 0.24 ^a^	39.7 ± 0.13 ^b^	39.6 ± 0.22 ^b^	38.4 ± 0.33 ^b^
Color a* Value	5.4 ± 0.04 ^a^	5.7 ± 0.04 ^b^	5.5 ± 0.07 ^a^	5.3 ± 0.06 ^a^
Color b* Value	20.1 ± 0.15 ^a^	20.3 ± 0.12 ^a^	20.1 ± 0.20 ^a^	19.2 ± 0.25 ^b^
Color C* Value	20.7 ± 0.21	20.8 ± 0.18	20.8 ± 0.20	19.9 ± 0.21
Color h° Value	74.5 ± 0.02	74.4 ± 0.08	74.7 ± 0.07	74.6 ± 0.05
Color ∆E	─	2.0 ± 0.07 ^a^	2.0 ± 0.02 ^a^	3.4 ± 0.23 ^b^

^a^, ^b^, ^c^—Values in the same row marked with different letters are significantly (α = 0.05) different. L*—lightness/darkness, a*—redness (+)/greenness (−), and b*—blueness (−)/yellowness (+), C*—chroma, h°—hue angle.

**Table 3:** Results of sensory and calorific evaluation of low-carbohydrate bread supplemented with poppy flour.

Addition of Poppy Flour (%)	Taste	Odour	Color	Texture	Overall Acceptability
0	5.6 ± 0.29 ^a^	5.6 ± 0.19	6.0 ± 0.34	5.3 ± 0.21 ^a^	5.7 ± 0.32 ^a^
5	5.9 ± 0.43 ^a^	6.0 ± 0.22	6.3 ± 0.31	5.3 ± 0.27 ^a^	5.9 ± 0.24 ^a^
10	5.8 ± 0.24 ^a^	5.8 ± 0.21	6.3 ± 0.23	5.3 ± 0.21 ^a^	5.9 ± 0.29 ^a^
15	5.3 ± 0.31 ^b^	5.7 ± 0.17	6.0 ± 0.21	4.7 ± 0.17 ^b^	5.4 ± 0.25 ^b^
	**Fat (%)**	**Fibre (%)**	**Carbohydrates (%)**	**Protein (%)**	**Energy (kcal/100g)**
0	3.3 ± 0.05 ^a^	12.3 ± 0.10 ^a^	16.9 ± 0.10 ^a^	17.5 ± 0.03 ^a^	192.3 ^a^
10	4.0 ± 0.09 ^b^	10.2 ± 0.09 ^b^	14.6 ± 0.12 ^b^	16.3 ± 0.08 ^b^	176.9 ^b^

^a^, ^b^—Values in the same column marked with different letters are significantly (α = 0.05) different.

**Table 4:** TPC and AA of flour and bread samples.

Sample	TPC [mg GAE/g d.m.]	EC_50_ ABTS [mg d.m./mL]	EC_50_ DPPH [mg d.m./mL]
Control Flour	1.45 ± 0.13 ^d^	34.6 ± 2.03 ^a^	85.2 ± 5.28 ^a^
Poppy Flour	0.42 ± 0.06 ^c^	204.1 ± 15.61 ^e^	211.3 ± 18.72 ^e^
0%	1.29 ± 0.15 ^b^	40.4 ± 3.26 ^b^	97.0 ± 4.31 ^b^
5%	1.21 ± 0.11 ^b^	44.7 ± 2.89 ^bc^	101.5 ± 6.13 ^bc^
10%	1.19 ± 0.09 ^ab^	48.2 ± 3.03 ^cd^	105.7 ± 5.97 ^c^
15%	1.05 ± 0.06 ^a^	50.9 ± 4.16 ^d^	116.9 ± 6.07 ^d^

^a^, ^b^, ^c^, ^d^, ^e^—Values in the same row marked with different letters are significantly (α = 0.05) different.

**Table 5:** Amino acid composition of control low-carbohydrate bread and bread with 10% PF.

Amino Acids	Amount of Amino Acid (mg∙g^−1^)	
	Control (0%PF)	Bread with 10% PF
Asparagine	**36.4 ± 0.85**	**38.1 ± 0.99**
Threonine	13.1 ± 0.25	13.5 ± 0.30
Serine	**17.6 ± 0.71**	**18.4 ± 0.68**
Glutamic Acid	**63.5 ± 1.39**	**67.0 ± 1.10**
Proline	**18.5 ± 0.37**	**19.2 ± 0.38**
Glycine	**16.1 ± 0.31**	**16.6 ± 0.24**
Alanine	**15.7 ± 0.46**	**16.8 ± 0.61**
Cysteic Acid	6.2 ± 0.23	5.9 ± 0.30
Valine	**15.6 ± 0.44**	**18.4 ± 0.46**
Methionine Sulfone	5.8 ± 0.38	5.7 ± 0.43
Isoleucine	14.3 ± 0.31	14.6 ± 0.39
Leucine	**23.5 ± 0.53**	**24.3 ± 0.64**
Tyrosine	9.3 ± 0.19	9.7 ± 0.21
Phenylalanine	17.2 ± 0.41	17.4 ± 0.48
Histidine	8.5 ± 0.31	8.7 ± 0.35
Lysine	20.1 ± 0.42	20.6 ± 0.41
Arginine	31.4 ± 0.87	32.1 ± 0.95
Tryptophan	**8.7 ± 0.32**	**7.3 ± 0.41**

Values in bold in the same row are significantly (α = 0.05) different.

**Table 6:** Fatty acid composition of control low-carbohydrate bread and bread with 10% PF.

Fatty Acids	Amount of Fatty Acids (g/100 g)	
	Control (0%PF)	Bread with 10% PF
Caprylic Acid (C8:0)	0.01 ± 0.00	0.01 ± 0.00
Capric Acid (C10:0)	0.01 ± 0.00	—
Lauric Acid (C12:0)	0.03 ± 0.01	0.01 ± 0.00
Myristic Acid (C14:0)	0.02 ± 0.00	0.01 ± 0.00
*cis*-9-Tetradecenoic Acid (C14:ln5)	—	—
Pentadecanoic Acid (C15:0)	0.002 ± 0.00	0.003 ± 0.00
Palmitic Acid (C16:0)	**0.39 ± 0.01**	**0.65 ± 0.01**
*cis*-9-Hexadecenoic Acid (C16:ln7)	0.01 ± 0.00	0.02 ± 0.00
Heptadecanoic Acid (C17:0)	0.003 ± 0.00	0.01 ± 0.00
*cis*-10-heptadecanoic Acid (C17:ln7)	0.001 ± 0.00	0.003 ± 0.00
Octadecanoic Acid (C18:0)	0.15 ± 0.01	0.23 ± 0.01
Oleic acid (C18:1n9c) + elaidic Acid (C18:1n9t)	1.51 ± 0.01	1.71 ± 0.01
Linoleic acid (C18:2n6c) + trans-9,12-Octadecadienoic Acid (C18:2n6t)	**1.01 ± 0.00**	**2.84 ± 0.01**
α-Linolenic Acid (C18:3n3(alpha))	1.36 ± 0.02	1.24 ± 0.01
Eicosanoic Acid (C20:0)	0.02 ± 0.00	0.03 ± 0.00
*cis*-11-Eicosenoic Acid (C20:1n9)	0.04 ± 0.00	0.03 ± 0.00
*cis*-11,14-Eicosadienoic Acid (C20:2n6)	0.003 ± 0.00	0.003 ± 0.00
Heneicosanoic Acid (C21:0)	0.001 ± 0.00	0.002 ± 0.00
cis-11,14,17-Eicosatrienoic Acid (C20:3n3)	0.001 ± 0.00	—
Behenic Acid (C22:0)	0.024 ± 0.01	0.026 ± 0.01
Erucic Acid (C22:1n9)	0.005 ± 0.00	0.005 ± 0.00
*cis*-13,16-Docosadienoic Acid (C22:2n6)	0.001 ± 0.00	—
Tricosanoic Acid (C23:0)	0.002 ± 0.00	0.003 ± 0.00
Lignoceric Acid (C24:0)	0.02 ± 0.00	0.02 ± 0.00
*cis*-15-tetracosenoic Acid (C24:1n9)	—	—
SFA (Saturated Fatty Acid)	**0.67 ± 0.02**	**0.97 ± 0.02**
MUFA (Mono Unsaturated Fatty Acid)	**1.56 ± 0.04**	**1.76 ± 0.03**
PUFA (Poly Unsaturated Fatty Acid)	**2.38 ± 0.06**	**4.10 ± 0.04**
OMEGA 3	**1.37 ± 0.05**	**1.23 ± 0.05**
OMEGA 6	**1.01 ± 0.07**	**2.85 ± 0.05**
OMEGA 9	**1.55 ± 0.05**	**1.74 ± 0.05**

Values in bold in the same row are significantly (α = 0.05) different.

**Table 7:** Locations of the ATR / FTIR absorption bands maxima and association with the appropriate vibration for selected samples, in the spectra range of 3750–450 cm^−1^.

Maximum Position (cm^−1^)		Types and Origin of Vibrations
Control Bread	Poppy Flour and Bread with 5, 10, 15 % PF	
3279	3277	ν_st_.(–OH) and Intermolecular H-Bonded
3006	3003	ν_st_. (C–H) in CH_2_
2918	2919	
2850	2851	
2665	2671	Overtone
1737	1738	ν (C=O)
1705	-	
1638	1638	δ (O–H) and Amide I
1539	1534	Amide II
1453	1448	ν (C–C)
1407	1399 / 1373	δ (–CH_2_) + ν (COO)
1309	1309	ν (C–C)
1275	-	
1234	1235	Amide III
1153	1143	ν_m_ (C–O) and ν_m_ (C–C)
1095	1071	
1044	1015	ν (C–O–C)
941	-	ν (C=C) And Skeletal Vibrations in the Pyranose Ring and Vibrations in the α-1,4-glycoside and α-1,6-glycoside Bonds Present in the Starch Structure
913	-	
772	763	
712	692	
662	650	
598	598	
510	510	

ν—stretching, δ—deformation, st.—strong, m—medium.

## Data Availability

The data presented in this study are available on request from the corresponding author.

## Sample Availability

Samples of the compounds are available upon request from the authors.
